# The Postoperative Appearance of the Liver on Ultrasonography Following Hydatid Cyst Surgery

**DOI:** 10.1155/1990/52160

**Published:** 1990

**Authors:** Tankut Ilter, Ali Menteş

**Affiliations:** 1 Departments of Gastroenterology and Surgery Agean University Faculty of Medicine Izmir Turkey; 2 Department of Surgery Agean University Faculty of Medicine Bornova Izmir 35100 Turkey

## Abstract

The postoperative ultrasound pattern in 33 patients with previous surgery for unilocular hydatid disease of the liver was investigated. Each patient was submitted to liver scanning with 99mTc pertecnatate, as well as to real time ultrasound examination. The patients were divided into three groups according to the type of surgery performed, namely; omentopexy, introflexion and omentoplexy with introflexion. Postoperative liver scans revealed defects similar to those detected during initial diagnosis in 88% of the patients, regardless of the surgical procedure performed. On the contrary, ultrasonography gave separate specific patterns for each surgical procedure.


HPB Surgery, 1990, Vol. 2, pp. 253-260
Reprints available directly from the publisher
Photocopying permitted by license only

© 1990 Harwood Academic Publishers GmbH
Printed in the United Kingdom

### THE POSTOPERATIVE APPEARANCE OF THE LIVER ON ULTRASONOGRAPHY FOLLOWING HYDATID CYST SURGERY

TANKUT ILTER and ALI MENTEŞ*

*Departments of Gastroenterology and Surgery, Agean University Faculty of Medicine, Izmir, Turkey*

*(Received 8 November 1989)*

The postoperative ultrasound pattern in 33 patients with previous surgery for unilocular hydatid disease of the liver was investigated. Each patient was submitted to liver scanning with 99mTc pertecnatate, as well as to real time ultrasound examination. The patients were divided into three groups according to the type of surgery performed, namely; omentopexy, introflexion and omentoplexy with introflexion. Postoperative liver scans revealed defects similar to those detected during initial diagnosis in 88% of the patients, regardless of the surgical procedure performed. On the contrary, ultrasonography gave separate specific patterns for each surgical procedure.

KEY WORDS: Liver, diagnosis, surgery; ultrasound, diagnosis; hydatid disease, diagnosis, surgery.

### INTRODUCTION

Echinococcal hydatid disease of the liver is endemic in Turkey. Hydatid cysts have been detected in 3.7% of all patients screened with ultrasound because of liver enlargement[1]. Liver scans have been used extensively for the diagnosis of this disease however, this has been replaced by ultrasound and computed tomography recently. Ultrasonography is the first choice diagnostic tool in the majority of patients due to its low cost and high availability.

The treatment of hydatid cysts is surgical. The surgical policy is to evacuate the cyst content and treat the residual cavity either by introduction of an omental flap (omentopexy) or by inversion of the adventitia into the cavity (introflexion) or both. Chaimoff, Lubin and Dintsman[2] have shown that, the defect in the liver scans caused by the parasitic lesion persists in the postoperative period. This finding has been consistent with our experience. Several of our patients had to be assured by intensive studies that they were in fact free of recurrent disease, in spite of the scintigraphic appearance. Ultrasound has been very useful in these patients.

This present study was planned in an attempt to clarify the ultrasonographic patterns following surgery for hydatic cysts of the liver.

### MATERIAL AND METHODS

The hospital files of 74 patients who had undergone surgery previously due to hydatid liver disease were reviewed. Those patients with multiple cysts, infected

*Correspondence and reprint requests to: Prof. Dr. Ali Menteş Department of Surgery, Agean University Faculty of Medicine, Bornova, 35100, Izmir, Turkey.

cysts, recurrent cysts, cysts that had led to significant complications such as frank intrabiliary rupture and patients with alveolar cysts were excluded. Forty one patients with unilocular cysts of the liver were invited for evaluation.

The liver scanning of the patients were performed with Selo Super Scanner DS 7, after intravenous injection of 3 mCi (111 mBq) 99mTc pertecnatate.

Ultrasonographic examinations were performed with Toshiba SAL 22A Real Time Ultrasound, using a 3,5 mHz linear probe.

Both examinations were performed while the examiner had no knowledge on the type of surgery performed to the individual patient. Surgical data was made available for evaluation only after the entire study was over.

### RESULTS

Eight patients with recurrence or suspicion of a recurrence were left out of the series. The remaining 33 patients were grouped according to the type of surgery performed. Group 1 consisted of 17 patients with omentopexy, Group 2 consisted of 12 patients with introflexion and Group 3 consisted of four patients in whom the residual cavity was treated with an omental flap after inversion of the adventitia (omentopexy + introflexion).

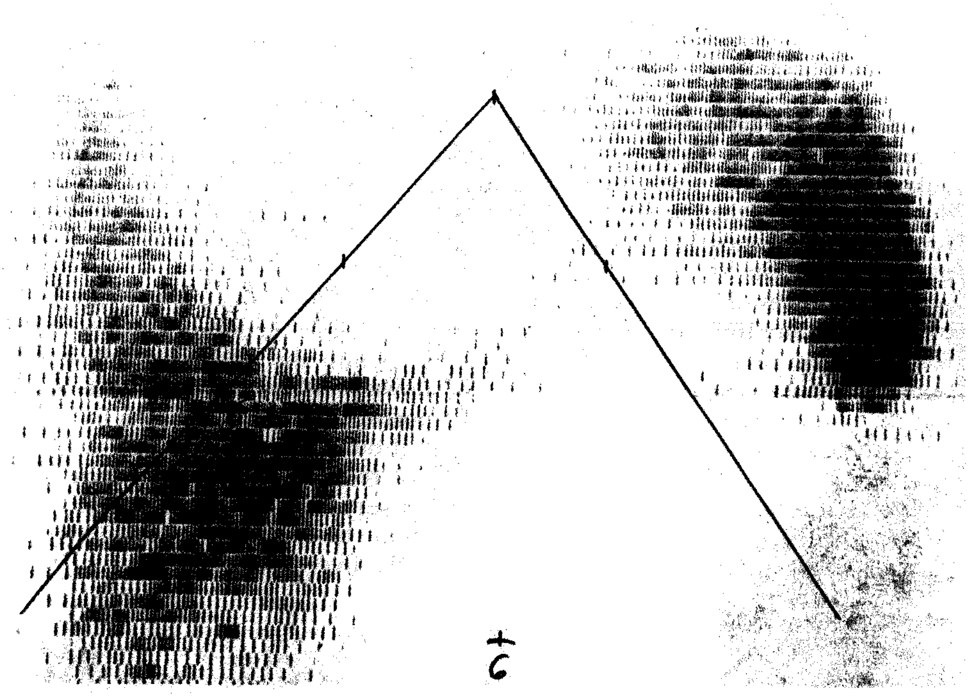

**Figure 1a** Preoperative liver scan of a patient with a hydatid cyst which occupies the entire left lobe, as well as a portion of the right lobe.

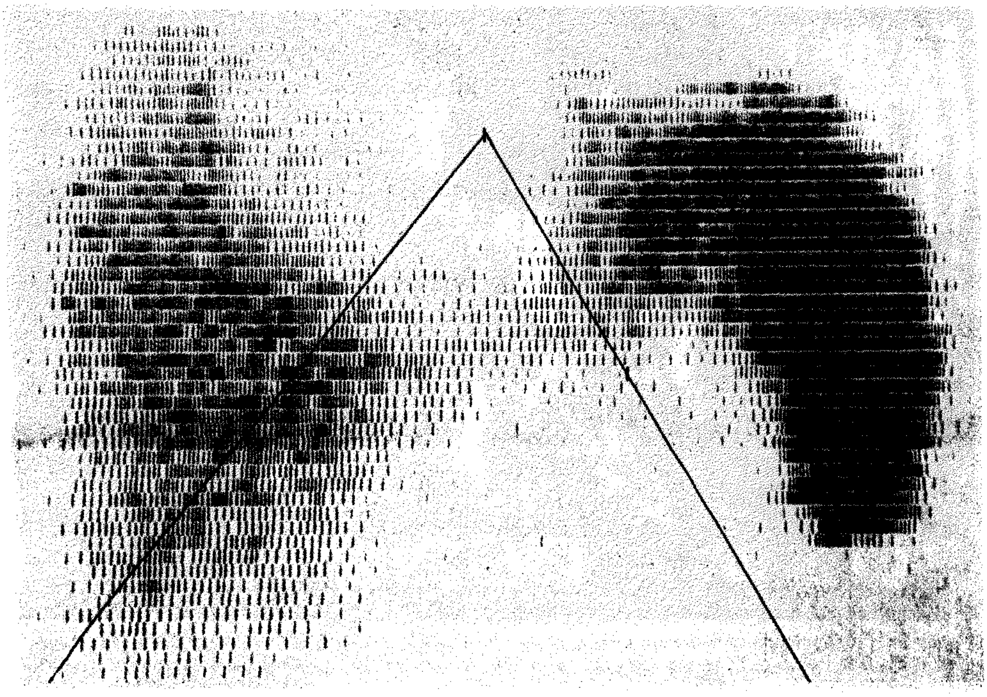

**Figure 1b** Late postoperative liver scan of the same patient following omentopexy. Note that the defect in (1a) persists almost entirely. This persistent scintigraphic defect was present in 94.1% of the patients after omentopexy and in 75% of those after introflexion.

*Group 1 (Omentopexy)*

The 99mTc liver scans revealed defects similar to those existing prior to surgery in 94.1% (16 out of 17) of the patients.

Ultrasound showed a smooth contoured, oval or round homogene mass with hyperechogenicity in all 17 patients (100%). An acoustic shadow could not be seen behind this mass. A hypoechogenic halo was present around the mass in 50% of the cases. The ultrasound appearance in these patients closely resembled a tumor (the "pseudotumor appearance"). The dimensions of the echogenic mass ranged from 4 × 4 cm.s to 8 × 8 cm.s, according to the size of the original cyst and in proportion to the thickness of the omental flap that had been introduced into the cavity.

*Group 2 (Introflexion)*

There was no abnormal finding on ultrasound of the liver in five out of 12 patients (41.7%) who had previously undergone an introflexion operation. In seven patients (58.3%) there was either a hypoechogenic or a hyperechogenic area at the site of the cyst in the liver. These areas had obscure contours and showed irregular vascularity. The size of these areas were between 3 × 4 and 6 × 6 cm.s and were hardly distinguished from the normal liver tissue.

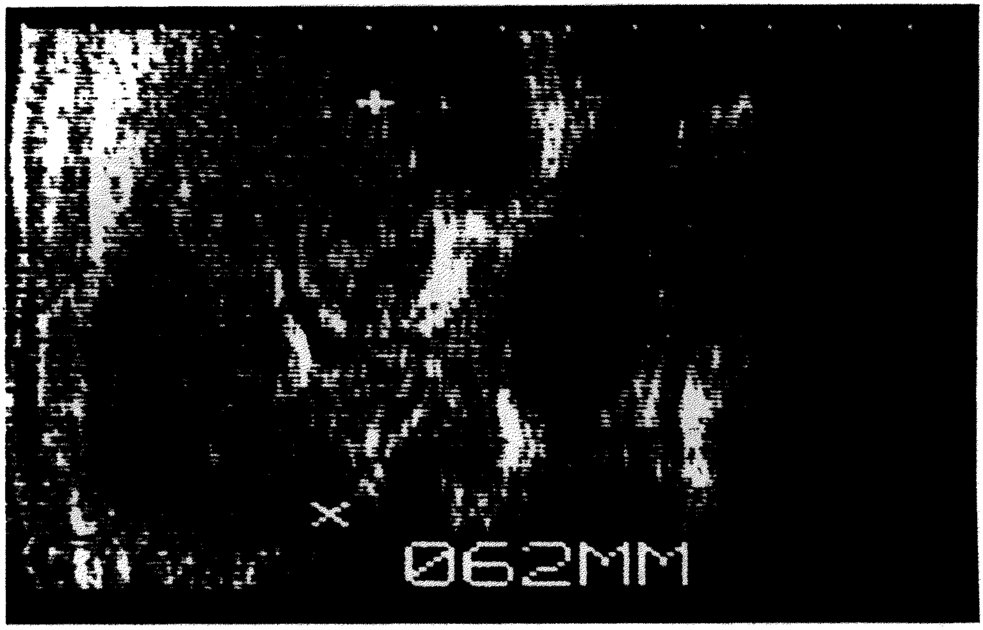

**Figure 2** Postoperative ultrasound picture of the liver following omentopexy for hydatid liver cyst. A round, smooth contoured, hyperechogenic mass within the liver structure is easily defined ("pseudotumor appearance"). An additional hypoechogenic hallo was present in 50% of the patients.

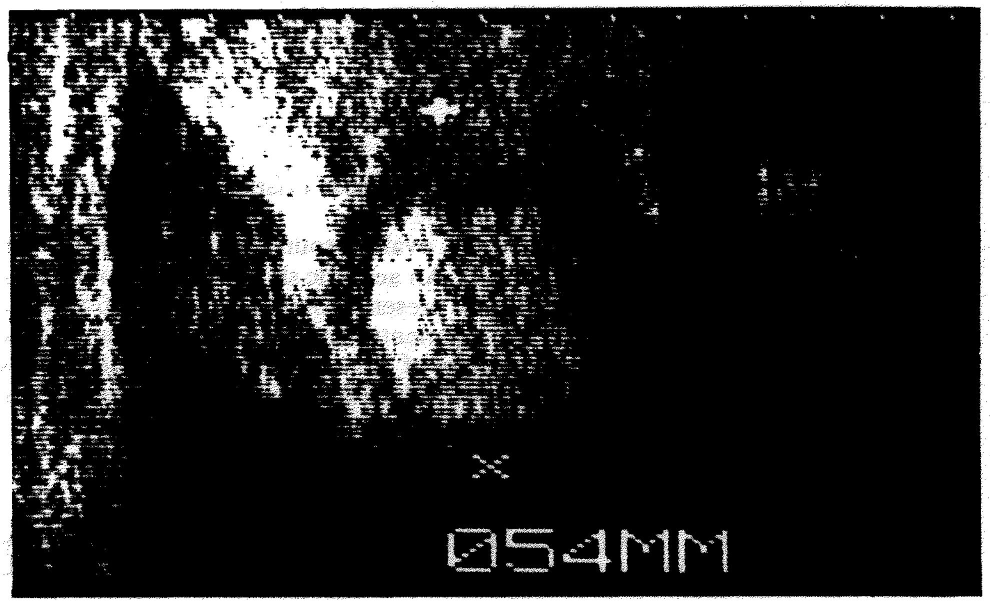

**Figure 3** Postoperative ultrasound appearance following introflexion. Obscure contours and irregular vascularity due to various degrees of fibrosis within the liver substance are distinctive from the smooth hyperechogenic mass of omentopexy.

In 40% (2/5 patients) of the cases with a normal ultrasound appearance and in all of the cases (7 patients) with irregularity on ultrasound, liver scans revealed marked hypoactivity in the area where the original cyst existed. Thus, a defect was persistently present in the liver scans in 75% of the patients who had undergone introflexion.

*Group 3 (Omentopexy + Introflexion)*

The four patients who comprised this group exhibited the combined postoperative ultrasonographic features detected both after omentopexy and introflexion. All four patients exhibited an area with irregular echogenity and vascularity, typical of introflexion. In addition, an echogenic spherical structure (one with and two without a surrounding halo) had been demonstrated in three of these patients.

Liver scans with 99mTc once again demonstrated inactivity at the site of the original cyst in these patients.

### DISCUSSION

The results obtained from this present study have confirmed the fact that, the neoplastic defects present in liver scans in patients with echinococcal cystic disease persist after surgery, irrespective of the kind of nonresective surgery performed. In this series 94% of the patients who had received omentopexy and 75% of those who received introflexion exhibited scintigraphic patterns similar to that obtained prior to surgery. The time elapse between surgery and the evaluation was 5 to 52 months in the omentopexy group and 5 to 61 months in the introflexion group. Hence, the scintigraphic findings did not seem to be related to the length of the follow up. Chaimoff, Lubin and Dintsman (2) have followed patients with omentopexy for hydatid cysts of the liver with liver scans for up to five years with similar findings.

Several authors have studied and described the classical ultrasonographic pattern of echinococcal cysts in detail[3,4]. It is quite easy to diagnose these lesions when they exhibit the usual signs. Diagnostic problems arise when the cyst is infected or when it has a solid appearance[5]. Cysts with the "ball of wool" or "yarn" sign[1] pose a similar problem. Ultrasound is again very helpful in evaluating patients with previous surgery for hydatid cysts. However, a detailed description of the postoperative ultrasound pattern has not been reported previously.

The results obtained in this present study have demonstrated that patients who have been treated with omentopexy or introflexion exhibit different ultrasonographic patterns after surgery. A smooth contoured, hyperechogenic, oval or round mass with or without a surrounding hypoechogenic halo is typical for omentopexy. The features that distinguish this lesion from a neoplastic growth are as follows: surgery for hydatid disease is evident from the medical history; the mass is well contoured; the echogenicity of the mass is homogenous and prominent; and the same appearance persists on repeated ultrasound examinations. Malignancy may be further excluded with fine needle aspiration cytology.

On the other hand, when introflexion is concerned, ultrasound reveals either a normal liver architecture or irregularity in terms of vascularity and echogenicity, in the area of the introflexed cyst.

One of the problems encountered during the postoperative ultrasound examination of the liver after surgery for hydatid cyst disease is related to the detection of the recurrences. The best policy is to evaluate the patient clinically as well as with the ultrasound findings. During the early postoperative period, some patients develop pseudocystic lesions secondary to surgery[6], which are resorbed in due course. This fact, as well as the natural course of the hydatid cyst, suggests that there must be a certain time lapse between surgery and the ultrasound examination before a definite decision can be made on whether the patient has a recurrence or a pseudocystic lesion. An exception to this policy may be the availability of an ultrasound profile of the liver immediately after surgery. In this study, the authors have accepted a 12 month period to decide whether the patient had a recurrence or not. Six out of eight patients matched this criteria and it was decided that these patients had recurrences. The period between surgery and follow up was five and seven months, respectively, in the other two patients. These patients are considered to have possible recurrences and have been put on careful periodic follow up.

*References*

1. Ilter, T., Özgüven, Ö. and Menteş, N.K., (1985) "Ball of Wool" or "Yarn" sign: A new ultrasonographic sign for the diagnosis of hydatid cysts. A preliminary report. *British Journal of Radiology*, **58**, 1141
2. Chaimoff, C., Lubin, E. and Dintsman, M. (1980) The postoperative appearance of the liver on scanning following omentopexy of the hydatid cyst, *International Surgery*, **65**, 331–333
3. Gharbi, H.A., Hassine, W., Brauner, M.W. and Dupuch, K. (1981) Ultrasound examination of the hydatid liver, *Radiology*, **139**, 459–463
4. Hadidi, A. (1982) Sonography of hepatic echinococcal cysts, *Gastrointestinal Radiology*, **7**, 349–354
5. Barriga, P., Cruz, F., Lepe, V. and Lathrop, R. (1983) An ultrasonographically solid, tumor-like appearance of echinococcal cysts of the liver, *Journal of Ultrasound Medicine*, **2**, 123–125
6. Weill, F., Le Mouel, A., Rohmer, P. and Bihr, E. (1981) Pseudo-cystic patterns after removal of hydatid cysts involving the liver, *European Journal of Radiology*, **1**, 238–240

(*Accepted by S. Bengmark on 8 November 1989*)

### INVITED COMMENTARY

Hydatid disease is a great worldwide medical problem, and is the most serious global tapeworm infection[1]. The most commonly affected organ is the liver, the second in frequency is hydatid disease of the lung. These organs are usually also involved in patients with hydatid disease elsewhere in the body. Multiple cysts are common. The disease is acquired in childhood, but usually not diagnosed before adulthood. Non-symptomatic hydatid disease is common, symptomatic disease can be due to cyst rupture or infection[2].

Although medical therapy for hydatid disease has been advocated, and promising results presented,[3] surgery remains the therapy of choice[2,4]. The cystic lesions contains scoleces, which represents the larval stage of the organism, and an antigenic transudate of serum, a clear cyst fluid. After operative cyst removal the pericyst, a fibrous capsule, remains. To reduce the size of the remaining cavity, internal suturing can be performed.

If it is not possible to effectively reduce the size of the remaining cavity in this way, packing of the cavity with omentum (omentopexy) is a preferred alternative[4].

In spite of these attempts to reduce the size of the cavity, follow-up liver scintigraphy will reveal a defect in the liver parenchyma in most patients[4,5]. It might be difficult to decide clinically if the patient has active disease, or if the defect seen only represents the remnant of previous surgery. Since information of the type of surgery performed is often lacking, a simple method with a higher degree of specificity than liver scintigraphy is needed for the post-operative evaluation in these patients. The radiological appearance of hydatid disease of the liver has been well described. Both ultrasonography and computed tomography have a high accuracy in detecting hydatid liver disease[1,2]. Ultrasound is suitable in these patients since it is easily performed and quite freely available. The presence of a liver cyst, with or without a thick wall, and especially when it contains a fluid-level or calcifications, should be regarded as highly suspect or hydatid disease in an endemic area. The presence of multiple daughter cysts, a separation of the laminated inner membrane from the wall of the cyst sometimes leading to a complete collapse of the same, and multiple infoldings of the inner cyst wall are all regarded as pathognomonic of hydatid disease[6]. The radiological appearance of hydatid disease outside the liver is the same. The differential diagnoses to consider are primarily other fluid-containing lesions like benign cysts, abscesses and resolving hematomas[6].

The radiological appearance of the liver after treatment of hydatid disease has also been described, both after medical[3] as well as after surgical treatment[4,5]. It has been noted that the liver, even a long time after surgery, does not return to a normal appearance, regardless of whether the patient has signs of active hydatid disease or not[4]. However, the number of reported cases have been few, and the surgical procedures performed varied. To the best of my knowledge, no previous material concerning the postoperative appearance of the liver after well defined surgical procedures for hydatid disease have been published.

In the present, valuable material the expected defect in the liver was seen in nearly all patients examined with liver scintigraphy.

However, with ultrasonography it was possible to notice a difference in the appearance of the liver depending on the type of surgery performed. All patients treated with omentopexy only, and three out of four treated with introflexion and omentopexy showed a roundish hyperechogenic lesion in the liver which represented the omentum introduced into the cavity.

In about half the patients a hypoechogenic halo was also seen around this lesion. On the contrary, in patients where no omentum had been introduced into the liver either had a normally appearing liver parenchyma or showed various distorsion of the liver echogenicity. No rounded, hyperechogenic lesion could be seen in any of these patients.

It is correctly remarked upon that a certain amount of time must pass between the surgery and the postoperative evaluation of the liver parenchyma. This is needed to allow resorption of a postoperative fluid accumulation that could otherwise be interpreted as recurrence of hydatid disease. It is also correctly noted that a correct medical history is needed to avoid misinterpretation of the findings at ultrasonography. However, this is not only the case in this group of patients. Regardless of the indication and method used, knowledge of previous surgical procedures greatly facilitates the correct interpretation of radiological examinations. If such information is given to the radiologist, the hyperechogenic area in the liver seen after omentopexy for hydatid disease can be correctly interpreted as an expected postoperative phenomenon. Thus, the patient can be spared unnecessary, costly and cumbersome examinations, and also escape much anxiety.

Christer Lundstedt
Dept. Diagn. Radiology
Lund University
LUND

*References*

1. Ali Hadidi (1982) Sonography of hepatic echinococcal cyst. *Gastrointest. Radiol.* **7**, 349–354
2. Beggs, I. (1985) Review: The radiology of hydatid disease. *Amer. J. Roentgenol.* **145**, 639–648
3. Morris, D.L., Skene-Smith, H., Haynes, A., Burrows, G.O. (1984) Abdominal hydatid disease. Computed tomographic and ultrasound changes during albendazole therapy. *Clin. Radiol.* **35**, 297–300
4. Beggs, I., Walmsley, K., Cowie, G.A. (1983) The radiological appearance of the liver after surgical removal of hydatid cyst. *Clin. Radiol.* **34**, 565–571
5. Chainoff, Ch., Lubin, E., Dintsman, M. (1980) The postoperative appearance of the liver in scanning following omentopexy of the hydatid cyst. *International Surgery* **65** (4), 331–333
6. Pant, C.S., Gupta, R.K. (1987) Diagnostic value of ultrasonography in hydatid disease in abdomen and chest. *Acta Radiol*, **28**, **Fasc. 6**, 743–745

